# Circumscription of the genus *Lepra*, a recently resurrected genus to accommodate the “*Variolaria”-*group of *Pertusaria* sensu lato (Pertusariales, Ascomycota)

**DOI:** 10.1371/journal.pone.0180284

**Published:** 2017-07-11

**Authors:** Xinli Wei, Imke Schmitt, Brendan Hodkinson, Adam Flakus, Martin Kukwa, Pradeep K. Divakar, Paul M. Kirika, Jürgen Otte, Anjuli Meiser, H. Thorsten Lumbsch

**Affiliations:** 1 State Key Laboratory of Mycology, Institute of Microbiology, Chinese Academy of Sciences, Beijing, China; 2 Senckenberg Biodiversity and Climate Research Centre (BiK-F), Senckenberganlage 25, Frankfurt am Main, Germany; 3 Department of Biological Sciences, Institute of Ecology, Evolution and Diversity, Goethe Universität, Frankfurt am Main, Germany; 4 Squamules Unlimited, Henrico, VA, United States of America; 5 Laboratory of Lichenology, W. Szafer Institute of Botany, Polish Academy of Sciences, Lubicz 46, Kraków, Poland; 6 Department of Plant Taxonomy and Nature Conservation, University of Gdańsk, WitaStwosza 59, Gdańsk, Poland; 7 Departamento de Biología Vegetal II, Facultad de Farmacia, Universidad Complutense, Plaza de Ramon y Cajal s/n, Madrid, Spain; 8 Botany Department, NationalMuseums of Kenya, Nairobi, Kenya; 9 Science & Education, The Field Museum, Chicago, Illinois, United States of America; University of Parma, ITALY

## Abstract

Pertusarialean lichens include more than 300 species belonging to several independent phylogenetic lineages. Only some of these phylogenetic clades have been comprehensively sampled for molecular data, and formally described as genera. Here we present a taxonomic treatment of a group of pertusarialean lichens formerly known as “*Pertusaria amara*-group”, “*Monomurata*-group”, or “*Variolaria*-group”, which includes widespread and well-known taxa such as *P*. *amara*, *P*. *albescens*, or *P*. *ophthalmiza*. We generated a 6-locus data set with 79 OTUs representing 75 species. The distinction of the *Variolaria* clade is supported and consequently, the resurrection of the genus *Lepra* is followed. Thirty-five new combinations into *Lepra* are proposed and the new species *Lepra austropacifica* is described from mangroves in the South Pacific. *Lepra* is circumscribed to include species with disciform ascomata, a weakly to non-amyloid hymenial gel, strongly amyloid asci without clear apical amyloid structures, containing 1 or 2, single-layered, thin-walled ascospores. Chlorinated xanthones are not present, but thamnolic and picrolichenic acids occur frequently, as well as orcinol depsides. Seventy-one species are accepted in the genus. Although the distinction of the genus from *Pertusaria* is strongly supported, the relationships of *Lepra* remain unresolved and the genus is tentatively placed in Pertusariales incertae sedis.

## Introduction

Molecular data had a major impact on our understanding of the evolution and phylogenetic relationships of lichen-forming fungi, and this resulted in a dramatic change of the classification over the last decades [1–7]. Pertusarialean fungi, traditionally placed in the family Pertusariaceae, mirror these changes. Over twenty years ago one of us (HTL) became interested in this group of fungi since the delimitation of genera, especially between the two major genera *Ochrolechia* and *Pertusaria*, was unclear. At that point morphological and chemical characters suggested a complex pattern but did not allow for a clear understanding of the phylogenetic relationships in this group of lichen-forming fungi [8–10].

Subsequent phylogenetic studies based on molecular data have shown that the group is polyphyletic and that the issues of distinguishing the main genera in the group, *Ochrolechia* and *Pertusaria*, were partly due to the fact that the large genus *Pertusaria* was highly polyphyletic [11–19]. These studies mainly included non-tropical species. Using a combination of morphological and chemical characters, the four major clades found within *Pertusaria* s. lat. can be characterized phenotypically [12, 16]. Two of the three clades, which are different from *Pertusaria* s. str., were subsequently recognized at generic level: *Gyalectaria* to accommodate species with gyalectoid ascomata forming a sister-group relationship to *Coccotrema* [12], and *Varicellaria* to accommodate species containing lecanoric acid as major extrolite, having disciform ascomata, strongly amyloid asci and non-amyloid hymenial gel, 1-2-spored asci with 1-layered, thick-walled ascospores [13]. However, the largest of the clades distantly related to *Pertusaria*, the *Variolaria* group [16] has not yet been treated in more detail by us.

Recently, a phylogenetic study of the group confirmed that the *Variolaria* group is distinct from *Pertusaria* and a new genus, *Marfloraea* was described to accommodate thirteen species of the group [19]. Subsequently, Hafellner [20] provided a detailed discussion of the taxonomy and nomenclature of the *Variolaria* group and showed that the description of this new genus was superfluous since several names are available for the group. He resurrected the genus *Lepra* with *Marfloraea* as synonym. This interpretation was followed in Buaruang et al. [21] and Lendemer & Harris [22]. These recent publications prompted us to address the circumscription of the genus *Lepra* using a data set of six loci, including four nuclear protein-coding genes and two ribosomal genes–nuclear large subunit (nuLSU) and mitochondrial small subunit (mtSSU) DNA. In addition to a larger sampling of molecular markers, we have also extended our taxon sampling to include additional, mainly tropical species to better understand the delimitation of this genus.

## Materials and methods

### Ethics statements

None of the collecting locations of the specimens used in this study are in natural conservation areas and hence no specific permissions were required for collecting samples. Our field studies did not involve any endangered or protected species.

### Taxon sampling

Forty-eight specimens of *Lepra* were included in the study (S1 Table). In addition sequences of the related genera *Ochrolechia* and *Varicellaria*, three species of Megasporaceae, and *Pertusaria* s. str. were included. Based on previous studies [12, 13,15], samples of *Pertusaria* s. str. were chosen as outgroup.

### DNA amplification and sequencing

Total genomic DNA was extracted from thallus fragments following the manufacturers’ instructions using the DNeasy Plant Mini Kit (Qiagen). We generated sequences of six loci, including nuclear large subunit (nuLSU), mitochondrial small subunit (mtSSU), and protein-coding loci, including the largest subunit of the RNA polymerase II gene (*RPB1*), the minichromosome maintenance complex component 7 (*MCM7*), elongation factor 1-α (*EF1*) and ribosome maturation factor *TSR1*, since they have been shown to be phylogenetically informative in molecular studies of fungi at this phylogenetic level [13, 15, 16, 23–26]. The PCR reactions were performed and primers were used as described previously [12, 24,26]. PCR products were sequenced using an ABI PRISM™ 3730 DNA Analyzer (Applied Biosystems). The sequences were assembled using SeqMan 7.1.0 (Lasergene) and conflicts edited manually.

### Sequence alignments and phylogenetic analyses

Sequences were aligned using ClustalW [27] in BioEdit 7.2.5 [28]. The program Gblocks v0.91b [29, 30] was used to remove regions of alignment uncertainty, using options for a “less stringent” selection on the Gblocks web server (http://molevol.cmima.csic.es/castresana/Gblocks_server.html). To test for phylogenetic congruence among loci, well-supported clades in single-gene ML trees were compared and assessed among individual topologies [31]. Each locus was subjected to a maximum likelihood (ML) analysis and clade support was tested using 1000 bootstrapping pseudoreplicates with RAxML-HPC BlackBox 8.2.6 [32] on the Cipres Science Gateway (http://www.phylo.org). Results were visualized with FigTree 1.4.2 (http://tree.bio.ed.ac.uk/software/figtree/). Individual single locus topologies were assessed for well-supported (>75%) conflict compared to the other single locus ML trees and combined if no conflict was observed [31, 33]. Since no conflicts were detected in the single-gene trees, a concatenated analysis was performed. Phylogenetic analyses of the concatenated dataset were performed using RAxML-HPC BlackBox 8.2.6[32] and MrBayes 3.2.6 [34, 35] on the Cipres Science Gateway (http://www.phylo.org; Miller et al. 2010). The model for each of the six single genes being used in the phylogenetic analysis was estimated using jModelTest-2.1.9[36, 37]. In the ML analysis, the GTR+G+I model was used as the substitution model with 1000 bootstrapping pseudoreplicates. The data was partitioned according to the different genes. For RPB1, MCM7, EF1 and TSR1 data were also partitioned by codon position. Two parallel Markov chain Monte Carlo (MCMC) runs were performed each using 8,000,000 generations and sampling every 1,000 steps. A 50% majority rule consensus tree was generated from the combined sampled trees of both runs after discarding the first 25% as burn-in. Chain mixing and convergence were evaluated in Tracer v1.5 considering effective sample size (ESS) values >200 as a good indicator. The tree files were visualized with FigTree 1.4.2 (http://tree.bio.ed.ac.uk/software/figtree/).

### Nomenclature

The electronic version of this article in Portable Document Format (PDF) in a work with an ISSN or ISBN will represent a published work according to the International Code of Nomenclature for algae, fungi, and plants, and hence the new names contained in the electronic publication of a PLOS article are effectively published under that Code from the electronic edition alone, so there is no longer any need to provide printed copies.

In addition, new names contained in this work have been submitted to MycoBank from where they will be made available to the Global Names Index. The unique MycoBank number can be resolved and the associated information viewed through any standard web browser by appending the MycoBank number contained in this publication to the prefix http://www.mycobank.org/MB/. The online version of this work is archived and available from the following digital repositories: PubMed Central, LOCKSS.

## Results and discussion

**Fig 1:**
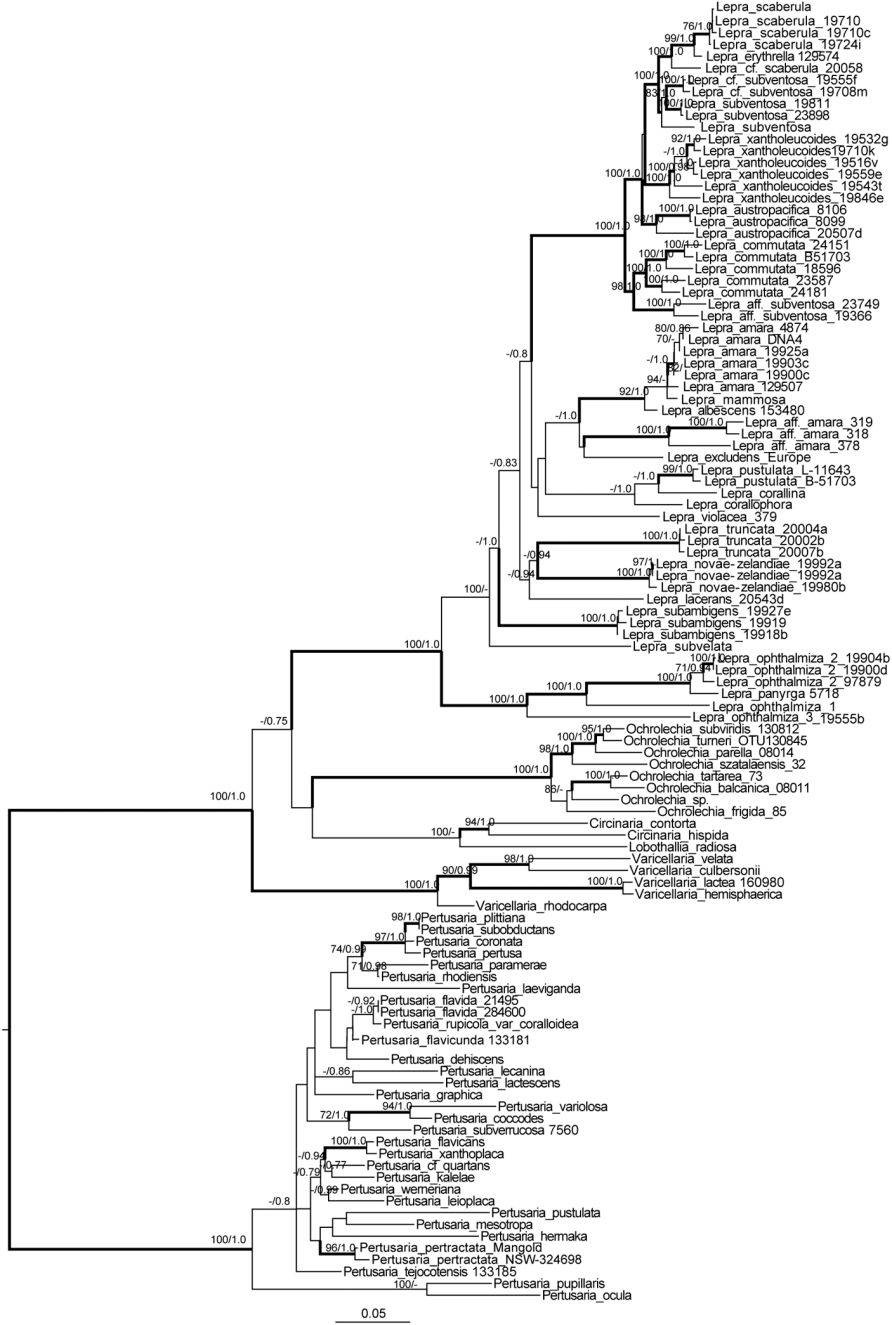
Phylogenetic relationships of *Lepra* and allied genera. This is a RAxML tree based on a concatenated 6-locus data matrix. The numbers above each node represent bootstrap support and posterior probability values, respectively, only values higher than 50% shown. Strongly supported nodes in bold. Scale = 0.03 substitution per site.

A total of 262 sequences of 6 gene loci were newly generated for this study, including 45 EF1α, 47 nuLSU, 43 MCM7, 48 mtSSU, 46 RPB1 and 33 TSR1 sequences. These were deposited in GenBank under accession numbers MF109133-MF109227, MF189726-MF189859and MF279153-MF279187 (S1 Table). In total, sequences of 177 samples were included in the study. The datasets used for this study were deposited in TreeBase (ID#20828). Single-locus maximum likelihood (ML) topologies are shown in the supplementary material (S1–S6 Figs). Since the ML and Bayesian trees of the concatenated, 6-locus data set (4325bp; nuLSU: 730bp, mtSSU: 741bp, RPB1: 598bp, MCM7: 554bp, EF1α: 980bp, TSR1: 722bp) were similar in their topology, only the ML trees with the posterior probabilities of the Bayesian analysis added is shown in Fig 1.

Our extended analysis including additional species and based on a six-locus dataset largely confirmed that the *Variolaria* group is distinct from *Pertusaria* s. str. and from the other two major clades that are now accepted as *Gyalectaria* and *Varicellaria*, respectively [12, 13]. As pointed out by Hafellner [20] the name *Lepra* is the oldest available name for this clade and is accepted here. Below we list all species that are currently included in this genus. The circumscription of *Lepra* largely agrees with the characterization of the *Variolaria* group described earlier [16]. The genus includes species with disciform ascomata, a weakly to non-amyloid hymenial gel, strongly amyloid asci without clear apical amyloid structures, single-layered, thin-walled ascospores. However, we here extend it to include species with single- and 2-spored asci, whereas we originally only included species with single-spored asci. Chlorinated xanthones are not present in species of *Lepra*, but thamnolic and picrolichenic acids occur in the genus, as well as orcinol depsides. Thamnolic acid is not restricted to this genus in the Pertusariales and additional studies are necessary to evaluate whether picrolichenic acid occurs outside *Lepra*. Species with 8-spored asci containing picrolichenic acid, such as *P*. *truncata* [38, 39], need further studies and are ad interim left in *Pertusaria* until we have a better understanding on the phylogenetic importance of depsones.

Our extended sampling with six loci did not result in a better support for the relationships of the different clades in Pertusariales and the sister-group relationship to a clade including *Ochrolechia* and Megasporaceae lacked support. Although the clade of *Varicellaria*, Megasporaceae, *Ochrolechia*, and *Lepra* was strongly supported as is each of these groups, the relationships among these four clades lacked support. Hence we propose to classify *Lepra* in Pertusariales inc. sed. until additional data become available with which the classification at family level in the order can be addressed.

In the phylogenetic tree (Fig 1), species of *Lepra* form a strongly supported monophyletic clade. Within this clade *L*. *ophthalmiza* and *L*. *panyrga* form a strongly supported early diverging monophyletic lineage.

Remarkably, several species, as currently circumscribed, were not monophyletic in our study and for most samples we have not drawn taxonomic consequences, since more material will be necessary to address species delimitation. The interspecific relationships within the latter group lack support. The phylogenetic relationships in the *L*. *ophthalmiza/L*. *panyrga* clade are strongly supported and indicate that *L*. *ophthalmiza* as currently circumscribed is polyphyletic–North American samples form a strongly supported sister-group relationship to *L*. *panyrga*, and these form a strongly supported sister-group relationship to the European *L*. *ophthalmiza* sample, whereas a specimen from Kenya forms a well-supported sister group to this clade. In the major clade of *Lepra* species for which more than one sample was included mostly form strongly supported monophyletic groups, with few exceptions, including *L*. *amara*. Samples identified as *L*. *amara* s. lat. based on morphology and chemistry fell into different clades with Japanese specimens being only distantly related to *L*. *amara* s.str. In addition *L*. *mammosa* nested within *L*. *amara* s. str., indicating that the species delimitation in this group requires further studies. The Japanese specimens tentatively identified as *L*. aff. *amara* have1-spored asci and contain dihydropertusaric, neodihydromurolic, isomuronic acids, and are with or without picrolichenic acid. Additional material will be necessary to elucidate the identity of these specimens but they probably represent an undescribed species. Furthermore, *L*. *scaberula* and *L*. *subventosa* require additional studies to better understand their circumscription. Samples of the former from the South Pacific (S1 Table) are described here as a new species, *L*. *austropacifica*. In the saxicolous *L*. *subventosa*, specimens from India, Kenya, Bolivia, and Australia clustered together, but differed in their extrolites. The Indian specimen contained lichexanthone and lecanoric acid, whereas the Kenyan material contained planaic and 4-*O*-demethylplanaic acids. The Australian specimen contains lichexanthone, picrolichenic acid, and thamnolic acid and agrees with *Lepra subventosa* s. str., whereas the Bolivian material contained lichexanthone, barbatic acid, and hypothamnolic acid.

Our study also showed that *Pertusaria copiosa* specimens clustered with *Lepra commutata*. Hence the former is below reduced to synonymy with *L*. *commutata*. This synonymy has also been proposed earlier based on phenotypical characters [40] and is confirmed here. Also Archer and Elix [40] placed *P*. *coccopoda* and *P*. *moreliensis* into synonymy of *Lepra xantholeucoides*. Our data show that *P*. *kinigiensis*, which differs from *L*. *xantholeucoides* in lacking lichexanthone but is otherwise very similar, is an additional synonym.

## Taxonomy

***Lepra*** Scop.

Intr. Hist. Nat.: 79 (1777)

*Type*: *Lichen albescens* Huds. [neotype, designated by Dibben (1980: 38)] = *Lepra albescens* (Huds.) Hafellner.

*= Variolaria* Pers., Ann. Bot. Usteri 7: 23 (1794), non Bull., Hist. Champ. Fr. 1: 181 (1791)

*Type species*: *Variolaria discoidea* Pers. = *Lepra albescens* (Huds.) Hafellner.

The name *Variolaria* is currently rejected against *Pertusaria*. However, the type species is not congeneric with *Pertusaria* as confirmed in this study. The name, however, as pointed out by Kondratyuk et al. (2015) and Hafellner [20], is not available for the *Variolaria* clade since it is a younger homonym of *Variolaria* Bull. (Art. 53.1), and hence illegitimate.

*= Leproncus* Ventenat, Tabl. Règne.Vég. 2: 32 (1799)

*Type species*: *Lichen albescens* Huds. [neotype, designated by Dibben (1980: 38)] = *Lepra albescens* (Huds.) Hafellner.

*= Isidium* (Ach.) Ach., Method. Lich.: xxxiii, 136 (1803) ≡*Lichen* subsect. *Isidium*Ach., K. Vetensk.-Acad. NyaHandl.15: 247 (1794)

*Type species*: *Isidium corallinum* (L.) Ach.≡*Lepra corallina* (L.) Hafellner.

*= Pertusaria* sect. *Lecanorastrum* Müll. Arg., Flora 67: 268 (1884)

*Type species*: *Pertusaria commutata* Müll. Arg. (lectotype, selected here) ≡*Lepra commutata* (Müll. Arg.) I. Schmitt, Hodkinson & Lumbsch

*= Pertusaria* subg. *Monomurata* Archer, Biblioth. Lichenol. 53: 6 (1993)

*Type species*: *Pertusaria commutata* Müll. Arg. ≡*Lepra commutata* (Müll. Arg.) I. Schmitt, Hodkinson & Lumbsch.

*= Pertusaria* sect. *Digitatae* Archer, Biblioth. Lichenol. 53: 7 (1993)

*Type species*: *Pertusaria gymnospora* Kantvilas ≡*Lepra gymnospora* (Kantvilas) I. Schmitt, Hodkinson & Lumbsch.

= *Marfloraea* S.Y. Kondr., L. Lökös & Hur, in Kondratyuk et al., Stud. Bot. Hungar. 46: 103 (2015)

*Type species*: *Marfloraea amara* (Ach.) S.Y. Kondr., L. Lökös & Hur. ≡*Lepra amara* (Ach.) Hafellner.

Nomenclatural notes.–The nomenclature of *Lepra* and its synonyms have been discussed in detail by Hafellner [20] who showed that *Lepra* Scop. [41] is the oldest available name for the *Variolaria* clade. Although this generic name was originally published without mentioning of a single species included in the genus, the selection by Dibben (1980: 38) of *Pertusaria albescens*as the type of the genus was effectively a neotypification.

**Lepra acroscyphoides** (Sipman)I. Schmitt, Hodkinson & Lumbsch, ***comb*. *nov*.** (MB 820747) Basionym: *Pertusaria acroscyphoides* Sipman, Willdenowia 16: 281 (1986).

**Lepra albescens** (Huds.) Hafellner, Stapfia 104: 171 (2016) ≡*Lichen albescens* Huds., Fl. Angl. p. 445 (1762) ≡*Pertusaria albescens* (Huds.) M. Choisy & Werner, Cavanillesia 5: 165 (1932). ≡*Marfloraea albescens* (Huds.) S.Y. Kondr., L. Lökös & Hur, in Kondratyuk et al., Stud. Bot. Hungar. 46: 105 (2015).

**Lepra alterimosa** (Darb.) I. Schmitt, Hodkinson & Lumbsch, ***comb*. *nov*.** (MB 820748) Basionym: *Pertusaria alterimosa* Darb., Wiss. Erg. Schwed. Südp. Exped. IV, 11: 7 (1912).

**Lepra amara** (Ach.) Hafellner, Stapfia 104: 171 (2016) ≡*Variolaria amara* Ach., K. Vetensk.-Acad. NyaHandl. 30: 163 (1809) ≡*Pertusaria amara* (Ach.) Nyl., Bull. Soc. Linn. Normandie, ser. 2, 6: 288 (1872) ≡*Marfloraea amara* (Ach.) S.Y. Kondr., L. Lökös & Hur, in Kondratyuk et al., Stud. Bot. Hungar. 46: 105 (2015).

**Lepra amaroides** (H. Magn.) I. Schmitt, Hodkinson & Lumbsch, ***comb*. *nov*.** (MB 820749). Basionym: *Pertusaria amaroides* H. Magn., Acta HortiGothob. 18: 214 (1950).

**Lepra amnicola** (Elix & A.W. Archer) I. Schmitt, Hodkinson & Lumbsch, ***comb*. *nov*.** (MB 820750). Basionym: *Pertusaria amnicola* Elix & A.W. Archer, Mycotaxon 64: 18 (1997).

**Lepra andersoniae** (Lendemer) Lendemer & R.C. Harris, Bryologist 120: 186(2017). Basionym: *Pertusaria andersoniae* Lendemer (as “*andersonii*”), Opusc. Philolich. 6: 55 (2009).

**Lepra aspergilla** (Ach.) Hafellner, Stapfia 104: 171 (2016) ≡*Lichen aspergillus* Ach., Lich. Suec. Prodr.: 1798 (1799) ≡*Pertusaria aspergilla* (Ach.) J.R. Laundon, Taxon 41: 745 (1992) ≡*Marfloraea aspergilla* (Ach.) S.Y. Kondr., L. Lökös & Hur, in Kondratyuk et al., Stud. Bot. Hungar. 46: 105 (2015).

**Lepra barbatica** (A.W. Archer & Elix) I. Schmitt, Hodkinson & Lumbsch, ***comb*. *nov*.** (MB 820752). Basionym: *Pertusaria barbatica* A.W. Archer & Elix, in Archer, BiblthcaLichenol. 69: 178 (1997).

**Lepra borealis** (Erichsen) I. Schmitt, Hodkinson & Lumbsch, ***comb*. *nov*.** (MB 820753). Basionym: *Pertusaria borealis* Erichsen, Annls. Mycol., 37: 354 (1939).

**Lepra buloloensis** (A.W. Archer) I. Schmitt & Lumbsch, MycoKeys 23: 82 (2017) ≡*Pertusaria buloloensis* A.W. Archer, Mycotaxon 56: 388 (1995).

**Lepra caucasica** (Erichsen) Hafellner, Stapfia 104: 172 (2016) ≡*Pertusaria caucasica* Erichsen, Fedd. Rep. 35: 379 (1934).

**Lepra clarkeana** (A.W. Archer) I. Schmitt, Hodkinson & Lumbsch, ***comb*. *nov*.** (MB 820754). Basionym: *Pertusaria clarkeana* A.W. Archer, Mycotaxon 53: 280 (1995) = *Pertusaria confusa* A.W. Archer, Mycotaxon 41: 224 (1991), non *P*. *confusa* Bory, Mém. Soc. Linn. Paris 4: 595 (1826).

**Lepra commutata** (Müll. Arg) Lendemer & R.C. Harris, Bryologist 120: 186 (2017). Basionym: *Pertusaria commutata* Müll. Arg., Flora 67: 269 (1884). Syn.: *Pertusaria copiosa* Erichsen, Ann. Mycol. 39: 391 (1941).

**Lepra corallina** (L.) Hafellner, Stapfia 104: 172 (2016) ≡*Lichen corallina* L., Mant. Pl. 1: 13 (1767). ≡*Variolaria corallina* (L.)Ach., K. Vetensk.-Acad. Nyl Handl. 30: 161 (1809) ≡*Pertusaria corallina* (L.) Arn., Flora 49: 658 (1861) ≡*Marfloraea corallina* (L.) S.Y. Kondr., L. Lökös & Hur, in Kondratyuk et al., Stud. Bot. Hungar. 46: 105 (2015).

**Lepra corallophora** (Vain.) Hafellner, Stapfia 104: 173 (2016) ≡*Pertusaria corallophora* Vain., Résult. Vot. Belg. Lich.: 22 (1903) ≡*Marfloraea corallophora* (Vain.) S.Y. Kondr., L. Lökös & Hur, in Kondratyuk et al., Stud. Bot. Hungar. 46: 105 (2015).

**Lepra dactylina** (Ach.) Hafellner, Stapfia 104: 172 (2016) ≡*Lichen dactylinus* Ach., Lich. suec. prodr.: 89 (1798) ≡*Isidium dactylinum* (Ach.) Ach., Method. Lich.: 137 (1803) ≡*Pertusaria dactylina* (Ach.) Nyl., Acta Soc. Sci. fenn. 7: 447 (1863).

**Lepra erythrella** (Müll. Arg.) I. Schmitt, Hodkinson & Lumbsch, ***comb*. *nov*.** (MB 820756). Basionym: *Pertusaria erythrella* Müll. Arg., Bull. Herb. Boissier 1: 41 (1893).≡*Marfloraea erythrella* (Müll. Arg.) S.Y. Kondr., L. Lökös & Hur, in Kondratyuk et al., Stud. Bot. Hungar. 46: 105 (2015).

**Lepra excludens** (Nyl.) Hafellner, Stapfia 104: 172 (2016) ≡*Pertusaria excludens* Nyl., Flora 68: 296 (1885) ≡*Marfloraea excludens* (Nyl.) S.Y. Kondr., L. Lökös & Hur, in Kondratyuk et al., Stud. Bot. Hungar. 46: 105 (2015).

**Lepra flavovelata** (Elix & A.W. Archer) I. Schmitt, Hodkinson & Lumbsch, ***comb*. *nov*.** (MB 820757). Basionym: *Pertusaria flavovelata* Elix & A.W. Archer, Mycotaxon 53: 276 (1995).

**Lepra floridana** (Dibben) Lendemer & R.C. Harris, Bryologist 120: 186 (2017). Basionym: *Pertusaria floridana* Dibben, Publ. Biol. Geol., Milw. Publ. Mus. 5: 55 (1980).

**Lepra graeca** (Erichsen) Hafellner, Stapfia 104: 172 (2016) ≡*Pertusaria graeca* Erichsen, Rabenh. Krypt. Fl. ed. 2, 9, 5. Abt., 1: 526 (1936).

**Lepra gymnospora** (Kantvilas) I. Schmitt, Hodkinson & Lumbsch, ***comb*. *nov*.** (MB 820759). Basionym: *Pertusaria gymnospora* Kantvilas, Lichenologist 22: 292 (1990).

**Lepra lacerans** (Müll. Arg.) I. Schmitt, Hodkinson & Lumbsch, ***comb*. *nov*.** (MB 820760). Basionym: *Pertusaria lacerans* Müll. Arg., Flora 67: 270 (1884).

**Lepra leonina** (Stizenb.) I. Schmitt, Hodkinson & Lumbsch, ***comb*. *nov*.** (MB 820761). Basionym: *Pertusaria leonina* Stizenb., Ber. Tätigk. St. Gall. naturw. Ges.: 242 (1890).

**Lepra leucosora** (Nyl.) Hafellner, Stapfia 104: 172 (2016) ≡*Pertusaria leucosora* Nyl., Flora 60: 223 (1877).

**Lepra leucosorodes** (Nyl.) I. Schmitt, Hodkinson & Lumbsch ***comb*. *nov*.** (MB 820762). Basionym: *Pertusaria leucosorodes* Nyl., Acta Soc. Sci. fenn. 26 (10): 16 (1900).

**Lepra macloviana** (Müll. Arg.) I. Schmitt, Hodkinson & Lumbsch, ***comb*. *nov*.** (MB 820763). Basionym: *Pertusaria macloviana* Müll. Arg., Flora 67: 271 (1884).

**Lepra mammosa** (Harm.) Hafellner, Stapfia 104: 172 (2016) ≡*Pertusaria mammosa* Harm., Lich. De France 5: 1141 (1913) ≡*Marfloraea mammosa* (Harm.) S.Y. Kondr., L. Lökös & Hur, in Kondratyuk et al., Stud. Bot. Hungar. 46: 105 (2015).

**Lepra melanochlora** (DC.) Hafellner, Stapfia 104: 172 (2016) ≡*Isidium melanochlorum* DC., in Lamarck & de Candolle, Fl. franç., edn. 3, 2: 326(1805) ≡*Pertusaria melanochlora* (DC.) Nyl., Bull. Soc. linn. Normandie, sér.2, 6: 289 (1872).

**Lepra miscella** (A.W. Archer) I. Schmitt, Hodkinson & Lumbsch, ***comb*. *nov*.** (MB 820764). Basionym: *Pertusaria miscella* A.W. Archer, Mycotaxon 41: 232 (1991).

**Lepra monogona** (Nyl.) Hafellner, Stapfia 104: 173 (2016) ≡*Pertusaria monogona* Nyl., Bull. Soc. linn. Normandie, sér. 2, 6: 289 (1872).

**Lepra multipuncta** (Turner) Hafellner, Stapfia 104: 173 (2016) ≡*Variolaria multipuncta* Turner, Trans. Linn. Soc. London 9: 137 (1806) ≡*Pertusaria multipuncta* (Turner) Nyl., Lich. Scand.: 179 (1861).

**Lepra multipunctoides** (Dibben) Lendemer & R.C. Harris, Bryologist 120: 187 (2017). Basionym: *Pertusaria multipunctoides* Dibben, Publ. Biol. Geol., Milw. Publ. Mus 5: 59 (1980) ≡*Variolaria multipunctoides* (Dibben) Lendemer, Hodkinson & R.C. Harris, Mems. NY Bot. Garden 104: 88 (2013).

**Lepra nerrigensis** (A.W. Archer & Elix) I. Schmitt, A.W. Archer & Lumbsch, ***comb*. *nov*.** (MB 820766). Basionym: *Pertusaria nerrigensis* A.W. Archer & Elix, in Archer, Bibl. Lichenol. 69: 195 (1997).

**Lepra novae-zelandiae** (Szatala) I. Schmitt, A.W. Archer & Lumbsch**, *comb*.*nov*.** (MB 820767). Basionym: *Pertusaria novae-zelandiae* Szatala, Borbásia 1: 60 (1939).

**Lepra ocellata** (Körb.) Hafellner, Stapfia 104: 172 (2016) ≡*Pertusaria ocellata* Körb., Denkschr. Schles. Ges. Vaterl. Cultur: 235 (1853) ≡*Variolaria ocellata* (Körb.) Darb., Bot. Jahrb. Syst. 22: 627 (1897).

**Lepra ophthalmiza** (Nyl.) Hafellner, Stapfia 104: 173 (2016) ≡*Pertusaria multipuncta* var. *ophthalmiza* Nyl., Lich. Scand.: 180. (1861) ≡*Pertusaria ophthalmiza* (Nyl.) Nyl., Flora 48: 354 (1865) ≡*Variolaria ophthalmiza* (Nyl.) Darb. *in* Engler, Bot. Jahrb. 22: 628 (1897) ≡*Marfloraea ophthalmiza* (Nyl.) S.Y. Kondr., L. Lökös & Hur, in Kondratyuk et al., Stud. Bot. Hungar. 46: 105 (2015).

**Lepra ornatula** (Müll. Arg.) I. Schmitt, Hodkinson & Lumbsch, ***comb*. *nov*.** (MB 820768). Basionym: *Pertusaria ornatula* Müll. Arg., Flora 67: 270 (1884).

**Lepra panyrga** (Ach.) Hafellner,Stapfia 104: 173 (2016) ≡*Urceolaria panyrga* Ach., Method. Lich.: 146 (1803) ≡*Pertusaria panyrga* (Ach.) A. Massal., Framm. Lich.: 53 (1855) ≡*Marfloraea panyrga* (Ach.) S.Y. Kondr., L. Lökös & Hur, in Kondratyuk et al., Stud. Bot. Hungar. 46: 105 (2015).

**Lepra paratropica** (Q. Ren) I. Schmitt, Hodkinson & Lumbsch, ***comb*. *nov*.** (MB 820769). Basionym: *Pertusaria paratropica* Q. Ren, Telopea 16: 134 (2014).

**Lepra patellifera** (A.W. Archer) I. Schmitt & Lumbsch, MycoKeys 23: 82 (2017) ≡*Pertusaria patellifera* A.W. Archer, Mycotaxon 41: 237 (1991).

**Lepra pseudolactea** (Erichsen) Hafellner, Stapfia 104: 172 (2016) ≡*Pertusaria pseudolectea* Erichsen, Ann. Mycol. 39: 144 (1941).

**Lepra pulvinata** (Erichsen) Hafellner, Stapfia 104: 172 (2016) ≡*Pertusaria graeca* Erichsen, Rabenh. Krypt. Fl. ed. 2, 9, 5. Abt., 1: 573 (1936).

**Lepra pustulata** (Brodo & W.L. Culb.) Lendemer & R.C. Harris, Bryologist 120: 187 (2017). Basionym: *Haematomma pustulatum* Brodo & W.L. Culb., Bryologist 89: 203 (1987) ≡*Loxospora pustulata* (Brodo & W.L. Culb.) R.C. Harris *in* Egan, Bryologist 93: 217 (1990) ≡*Variolaria pustulata* (Brodo & W. L. Culb.) Lendemer, Hodkinson & R.C. Harris, Mems. NY Bot. Garden 104: 88 (2013).

**Lepra rugifera** (Müll. Arg.) I. Schmitt, Hodkinson & Lumbsch, ***comb*. *nov*.** (MB 820771). Basionym: *Pertusaria rugifera* Müll. Arg., Miss. Sci. Cap Horn, Lich.: 163 (1888).

**Lepra scaberula** (A.W. Archer) I. Schmitt, Hodkinson & Lumbsch, ***comb*. *nov*.** (MB 820772). Basionym: *Pertusaria scaberula* A.W. Archer, Mycotaxon 41: 240 (1991) ≡*Marfloraea scaberula* (A.W. Archer) S.Y. Kondr., L. Lökös & Hur, in Kondratyuk et al., Stud. Bot. Hungar. 46: 106 (2015).

**Lepra schaereri** (Hafellner) Hafellner, Stapfia 104: 172 (2016) ≡*Pertusaria schaereri* Hafellner, Stapfia 76: 155 (2001).

**Lepra scutellifera** (A.W. Archer & Elix) I. Schmitt, Hodkinson & Lumbsch, ***comb*. *nov*.** (MB 820773). Basionym: *Pertusaria scutellifera* A.W. Archer & Elix, Mycotaxon 50: 208 (1994).

**Lepra sejilaensis** (Q. Ren) I. Schmitt, Hodkinson & Lumbsch, ***comb*. *nov*.** (MB 820774). Basionym: *Pertusaria sejilaensis* Q. Ren, Telopea 16: 137 (2014).

**Lepra slesvicensis** (Erichsen) Hafellner, Stapfia 104: 172 (2016) ≡*Pertusaria slesvicensis* Erichsen, Fedd. Rep. 35: 391 (1934).

**Lepra sphaerophora** (Oshio) I. Schmitt, Hodkinson & Lumbsch, ***comb*. *nov*.** (MB 820775). Basionym: *Pertusaria sphaerophora* Oshio, J. Sci. Hiroshima Univ., ser B, div. 2, 12: 96 (1968).

**Lepra stalactiza** (Nyl.) Hafellner, Stapfia 104: 172 (2016) ≡*Pertusaria stalactiza* Nyl., Flora 57: 311 (1874).

**Lepra subcomposita** (Oshio) I. Schmitt, Hodkinson & Lumbsch, ***comb*. *nov*.** (MB 820776). Basionym: *Pertusaria subcomposita* Oshio, J. Sci. Hiroshima Univ., ser. B, div. 2, 12: 95 (1968).

**Lepra subventosa** (Malme) I. Schmitt & Lumbsch, MycoKeys 23: 82 (2017) ≡*Pertusaria subventosa* Malme, Ark. Bot. 28A: 7 (1936) ≡*Marfloraea subventosa* (Malme) S.Y. Kondr., L. Lökös & Hur, in Kondratyuk et al., Stud. Bot. Hungar. 46: 106 (2015).

**Lepra subviolacea** (Q. Ren) I. Schmitt, Hodkinson & Lumbsch, ***comb*. *nov*.** (MB 820777). Basionym: *Pertusaria subviolacea* Q. Ren, Telopea 16: 138 (2014).

**Leprasuperans**(Müll. Arg.) I. Schmitt, Hodkinson & Lumbsch, ***comb*. *nov*.** (MB 820778). Basionym: *Pertusaria superans* Müll. Arg., Flora 67: 209 (1928).

**Lepra teneriffensis** (Vain.) Hafellner, Stapfia 104: 172 (2016) ≡*Pertusaria teneriffensis* Vain., Kgl. Danske Vidensk. Selsk. Skr., Naturvid. Math. Afd. 8, 6 (3): 394 (1924).

**Lepra trachythallina** (Erichsen) Lendemer & R.C. Harris, Bryologist 120: 187 (2017). Basionym: *Pertusaria trachythallina* Erichsen *in* Degelius, Ark. Bot. 30A: 36 (1940) ≡*Variolaria trachythallina* (Erichsen) Lendemer, R.C. Harris & Hodkinson, Mems. NY Bot. Garden 104: 88 (2013).

**Lepra tropica** (Vain.)Lendemer & R.C. Harris, Bryologist 120: 188 (2017). Basionym: *Pertusaria tropica* Vain. *in* Hiern., Cat. Welwitsch. Afric. Pl. 2: 404 (1901).

**Lepra tuberculata** (Erichsen) Hafellner, Stapfia 104: 172 (2016) ≡*Pertusaria globulifera* var. *tuberculata* Erichsen, Verh. Bot. Ver. Prov. Brandenb. 71: 114 (1929) ≡*Pertusaria tuberculata* (Erichsen) Erichsen, Flechtenfl. Nordwestd.: 264 (1957).

**Lepra variabilis** (Elix & A.W. Archer) I. Schmitt, Hodkinson & Lumbsch, ***comb*. *nov*.** (MB 820781). Basionym: *Pertusaria variabilis* Elix & A.W. Archer, Telopea 112: 270 (2008).

**Lepra variolosa** (Kremp.) I. Schmitt, A.W. Archer & Lumbsch, ***comb*. *nov*.** (MB 820782). Basionym: *Pertusaria subvaginata* f. *variolosa* Kremp., Flora 49: 218 (1896). *Pertusaria variolosa* (Kremp.)Vain., Acta Soc Fauna Flora Fenn. 7 (1): 106 (1890).

**Lepra ventosa** (Malme) Lendemer & R.C. Harris, Bryologist 120: 175 (2017). Basionym: *Pertusaria ventosa* Malme, Ark. Bot. 28A: 7 (1936).

**Lepra verdonii** (A.W. Archer) I. Schmitt, Hodkinson & Lumbsch, ***comb*. *nov*.** (MB 820784). Basionym: *Pertusaria verdonii* A.W. Archer, Proc. Linn. Soc. NSW 113: 68 (1992).

**Lepra violacea** (Oshio) I. Schmitt, Hodkinson & Lumbsch, ***comb*. *nov*.** (MB 820785). Basionym: *Pertusaria violacea* Oshio, J. Sci. Hiroshima Univ., ser B, div. 2, 12: 92 (1968).

**Lepra waghornei** (Hult.) Lendemer & R.C. Harris, Bryologist 120: 175 (2017). Basionym: *Pertusaria waghornei* Hult., Hedwigia, 35: 191 (1893) ≡*Variolaria waghornei* (Hult) Darb. *in* Engler, Bot. Jahrb. 22: 628 (1897).

**Lepra wawreana** (A. Massal.) I. Schmitt, Hodkinson & Lumbsch, ***comb*. *nov*.** (MB 820787). Basionym: *Pertusaria wawreana* A. Massal., Mem. Imp. RealeIst. Veneto 10: 78 (1861).

**Lepra wirthii** (Elix & A.W. Archer)I. Schmitt, Hodkinson & Lumbsch, ***comb*. *nov*.** (MB 820788). Basionym: *Pertusaria wirthii* Elix & A.W. Archer, Telopea 15: 116 (2013).

**Lepra xantholeuca** (Müll. Arg.) I. Schmitt, A.W. Archer & Lumbsch, ***comb*. *nov*.** (MB 820789). Basionym: *Pertusaria xantholeuca* Müll. Arg., Proc. R. Soc. Edinb. 11: 402 (1882).

**Lepra xantholeucoides** (Müll. Arg.) I. Schmitt, A.W. Archer & Lumbsch, ***comb*. *nov*.** (MB 820790). Basionym: *Pertusaria xantholeucoides* Müll. Arg., Nuovo Giorn. Bot. Ital. 21: 357 (1889). Syn.: *Pertusaria coccopoda* Vain., Ann. Acad. Fenn., ser. A 6 (7): 32 (1915). *Pertusaria kinigiensis* A.W. Archer et al., Nova Hedwigia 88: 315 (2009). *Pertusaria moreliensis* B. de Lesd., Lich. Mexique: 18 (1914).

### New species

**Fig 2:**
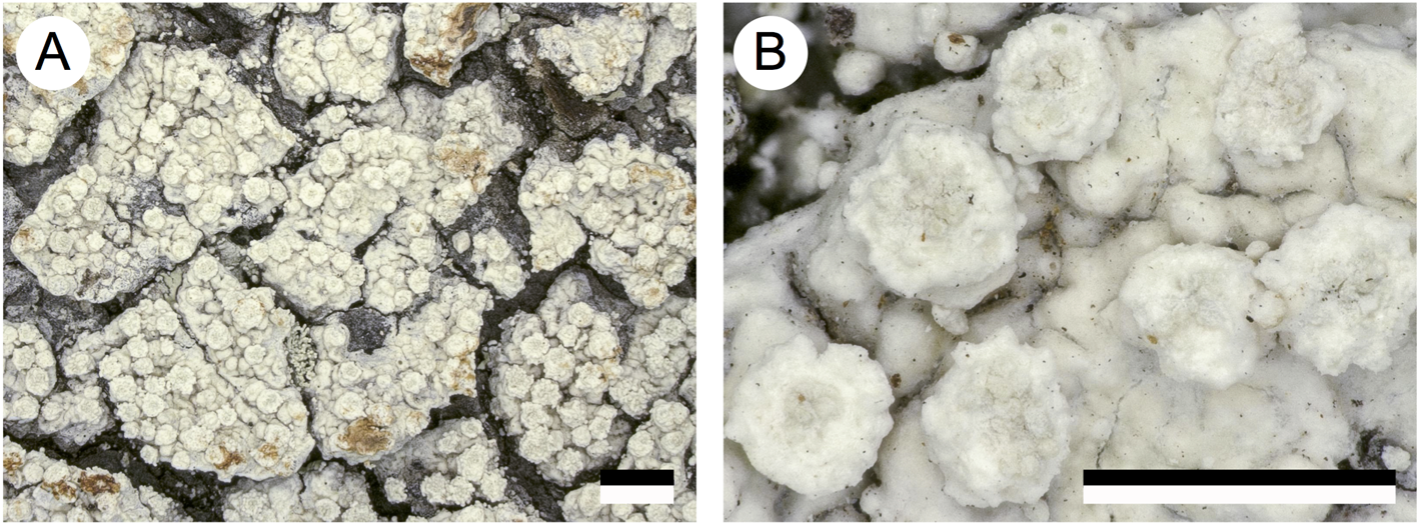
Morphology of the new species *Lepra austropacifica*, habit of the holotype. Scale = 1mm.

***Lepra austropacifica*** I. Schmitt & Lumbsch, ***sp*. *nov*.** [urn:lsid:mycobank.org:names:820791] (Fig 2)

Type: NEW CALEDONIA. Prov. Nord: Hienghene, Mon Paik, ca. 2km E of Hienghene, mangrove forest, 20°41’S, 164°57’E, 1 m alt., on bark, 10 August 2012, *K*. *Papong 8106 & H*.*T*. *Lumbsch* (holotype IRD; isotypes F & MSUT).

Diagnosis. Phenotypically similar to *Lepra scaberula* but differing in DNA sequence data.

Description. Thallus corticolous, crustose, verrucose to verruculose, thin, whitish to greyish white; margin indistinct; prothallus not visible; sorediate, lacking isidia. Soralia roundish, 0.5–1.5 mm diam., hemispherical, remaining distinct, with granular soredia, yellowish white to whitish grey. Apothecia and pycnidia not seen. Containing lichexanthone and thamnolic acid.

Etymology. Referring to the locality in the South Pacific.

Ecology and distribution. So far this corticolous species is only known from the South Pacific where it was found in mangrove forests and secondary forests at low elevations.

Additional specimens examined. NEW CALEDONIA. Prov. Nord: Hienghene, Mon Paik, ca. 2kmE of Hienghene, mangrove forest, 20°41’S, 164°57’E, 1 m alt., on bark, 10 August 2012, *K*. *Papong & H*.*T*. *Lumbsch 8099* (IRD); FIJI. VitiLevu: Suva area, Namosi Road, secondary forest along roadside, 18°03’S178°10’E, on fallen tree, October 2011, *H*.*T*. *Lumbsch 20507d* (F, SUVA).

Notes. The new species is very similar to *Lepra scaberula* and agrees with it in secondary chemistry. However, molecular data support its distinction. Separation of this sterile, cryptic species from *L*. *scaberula* without molecular data will be difficult.

## Supporting information

S1 Fig Phylogenetic relationships of *Lepra* and allied genera. This is a RAxML tree based on nuLSU DNA sequences. The numbers at each node represent bootstrap support value, and numbers lower than 50 not shown. Scale = 0.03 substitution per site.
(TIF)

S2 Fig Phylogenetic relationships of *Lepra* and allied genera. This is a RAxML tree based on mtSSU sequences. The numbers at each node represent bootstrap support value, and numbers lower than 50 not shown. Scale = 0.03 substitution per site.
(TIF)

S3 Fig Phylogenetic relationships of *Lepra* and allied genera. This is a RAxML tree based on RPB1 sequences. The numbers at each node represent bootstrap support value, and numbers lower than 50 not shown. Scale = 0.03 substitution per site.
(TIF)

S4 Fig Phylogenetic relationships of *Lepra* and allied genera. This is a RAxML tree based on MCM7 concatenated sequences. The numbers at each node represent bootstrap support value, and numbers lower than 50 not shown. Scale = 0.03 substitution per site.
(TIF)

S5 Fig Phylogenetic relationships of *Lepra* and allied genera. This is a RAxML tree based on EF1α sequences. The numbers at each node represent bootstrap support value, and numbers lower than 50 not shown. Scale = 0.03 substitution per site.
(TIF)

S6 Fig Phylogenetic relationships of *Lepra* and allied genera. This is a RAxML tree based on TSR1sequences. The numbers at each node represent bootstrap support value, and numbers lower than 50 not shown. Scale = 0.03 substitution per site.
(TIF)

S1 Table (DOCX)
