# Supplementary figures and images for: Circumscription of the genus Lepra, a recently resurrected genus to accommodate the “Variolaria”-group of Pertusaria sensu lato (Pertusariales, Ascomycota)
Source: PLoS One. 2017 Jul 11;12(7):e0180284. doi: 10.1371/journal.pone.0180284 (PMC5507398; doi:10.1371/journal.pone.0180284)

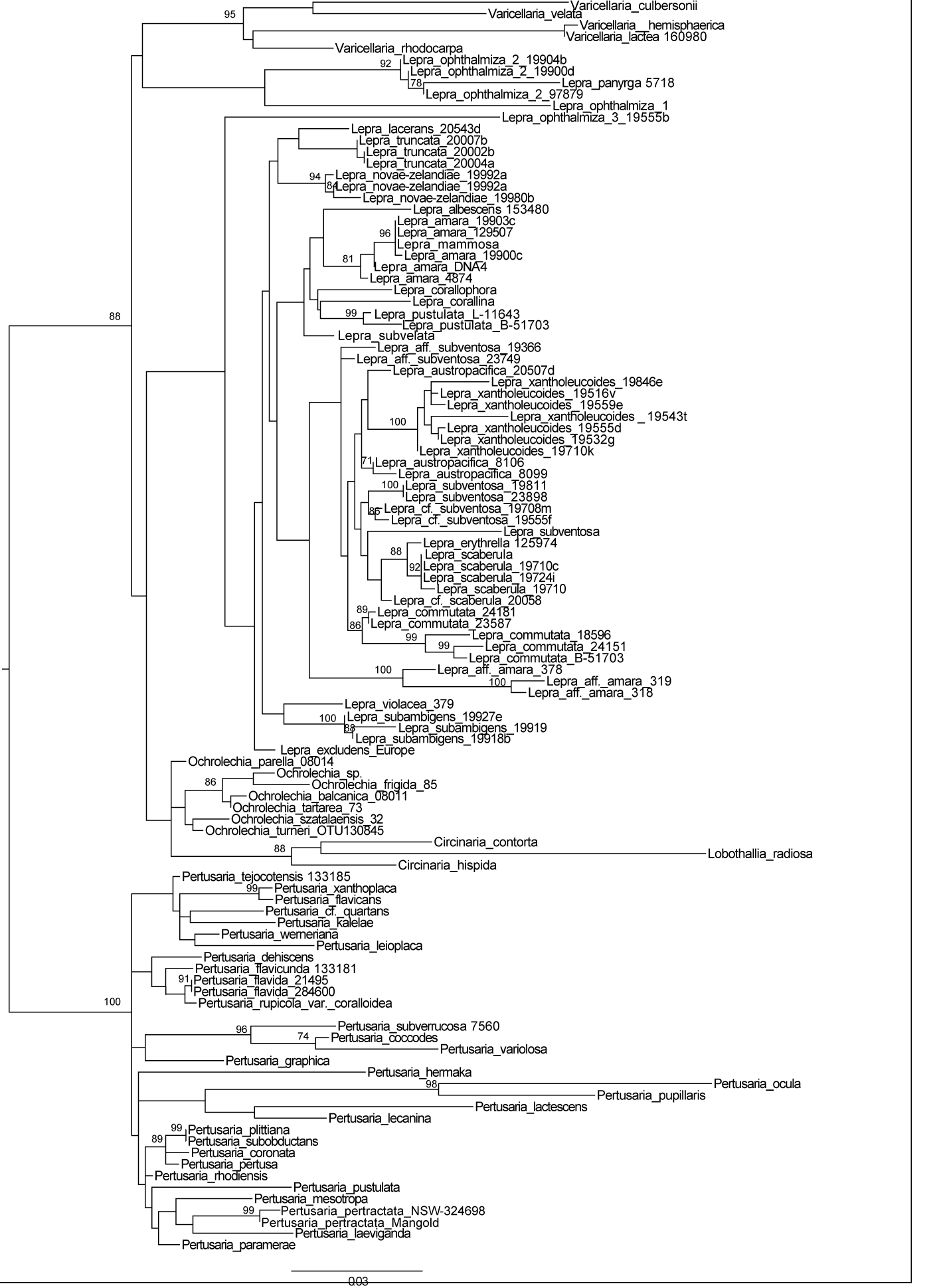

Supplement: S1 Fig — This is a RAxML tree based on nuLSU DNA sequences. The numbers at each node represent bootstrap support value, and numbers lower than 50 not shown. Scale = 0.03 substitution per site. (TIF) [file pone.0180284.s001.tif]

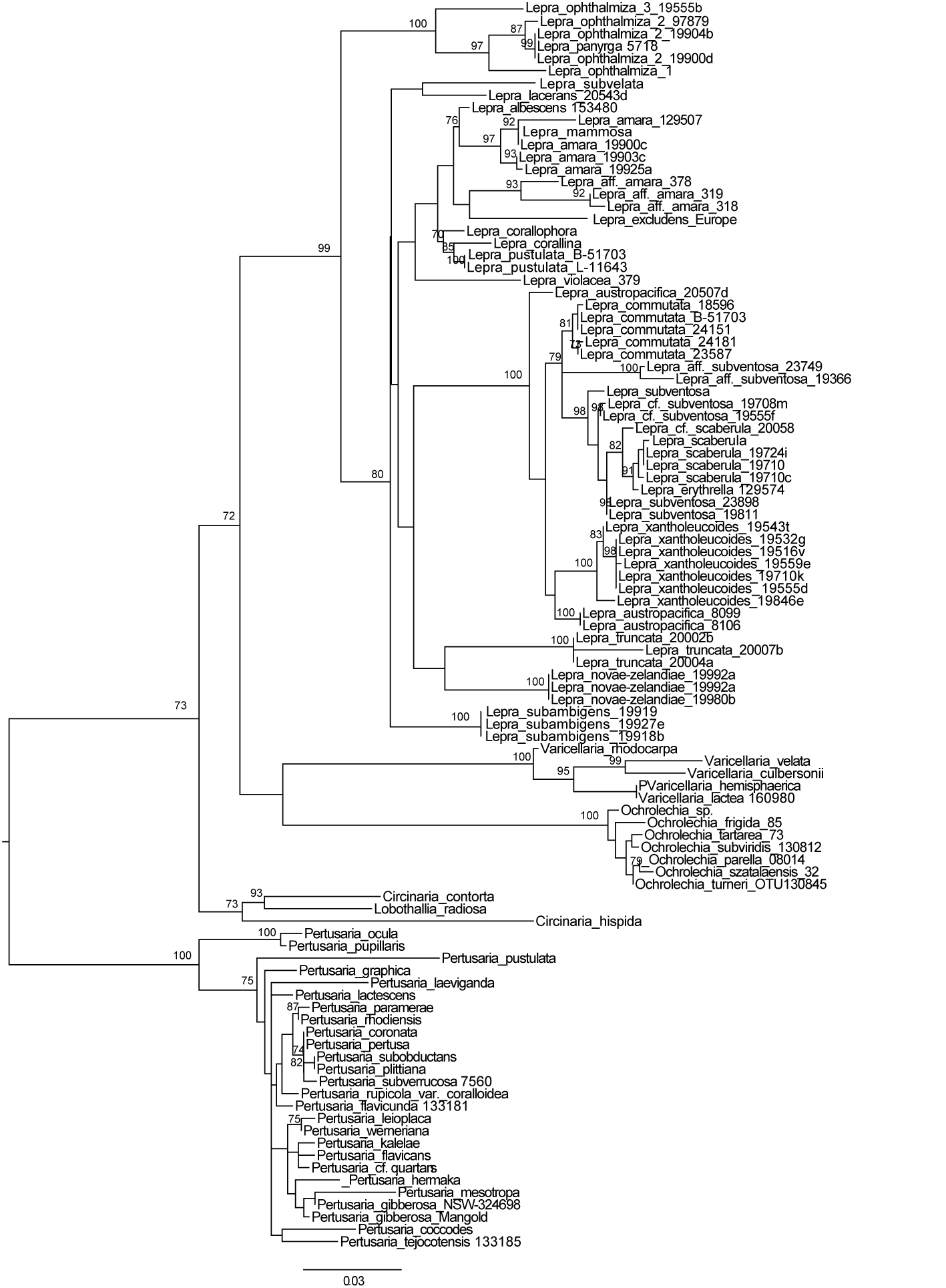

Supplement: S2 Fig — This is a RAxML tree based on mtSSU sequences. The numbers at each node represent bootstrap support value, and numbers lower than 50 not shown. Scale = 0.03 substitution per site. (TIF) [file pone.0180284.s002.tif]

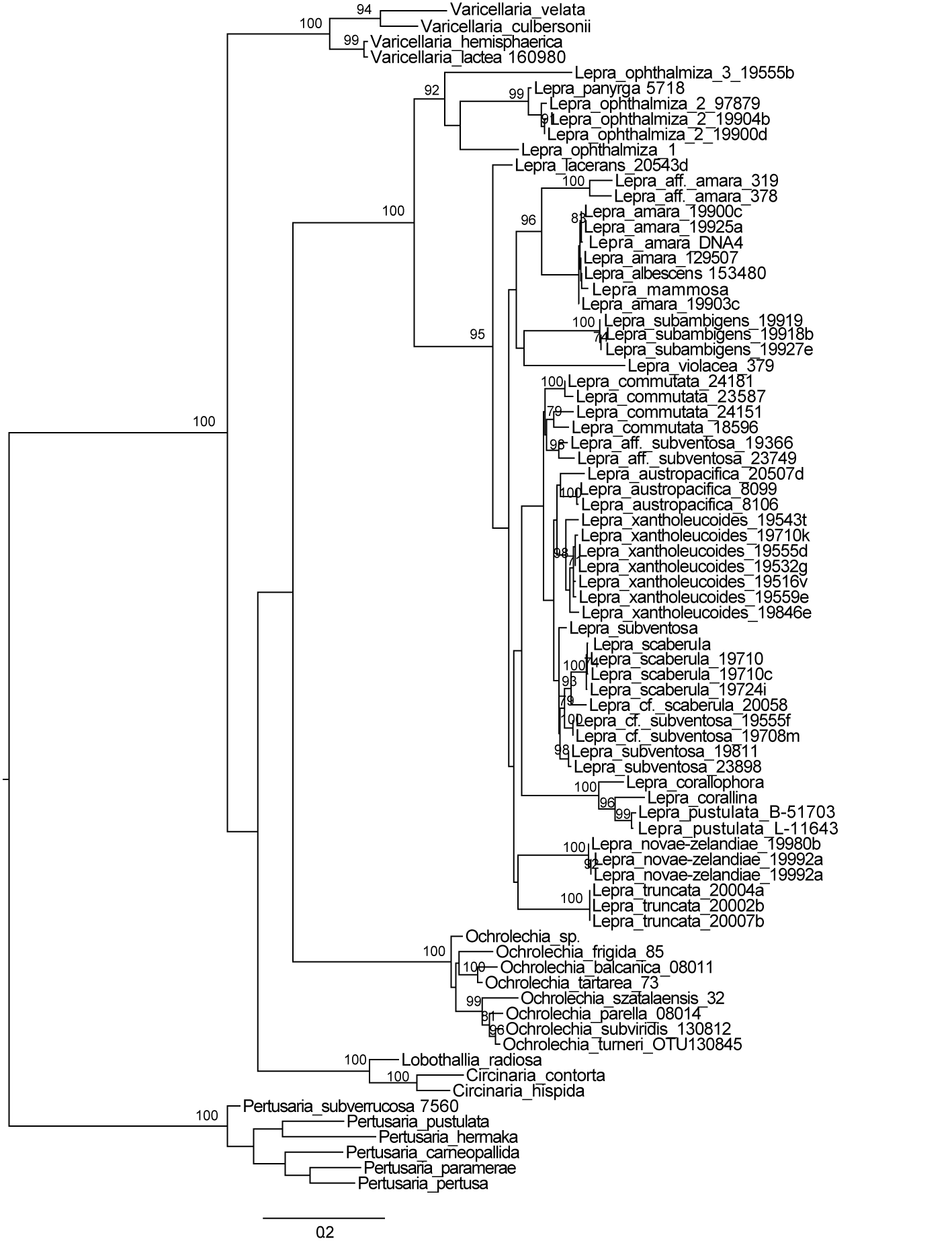

Supplement: S3 Fig — This is a RAxML tree based on RPB1 sequences. The numbers at each node represent bootstrap support value, and numbers lower than 50 not shown. Scale = 0.03 substitution per site. (TIF) [file pone.0180284.s003.tif]

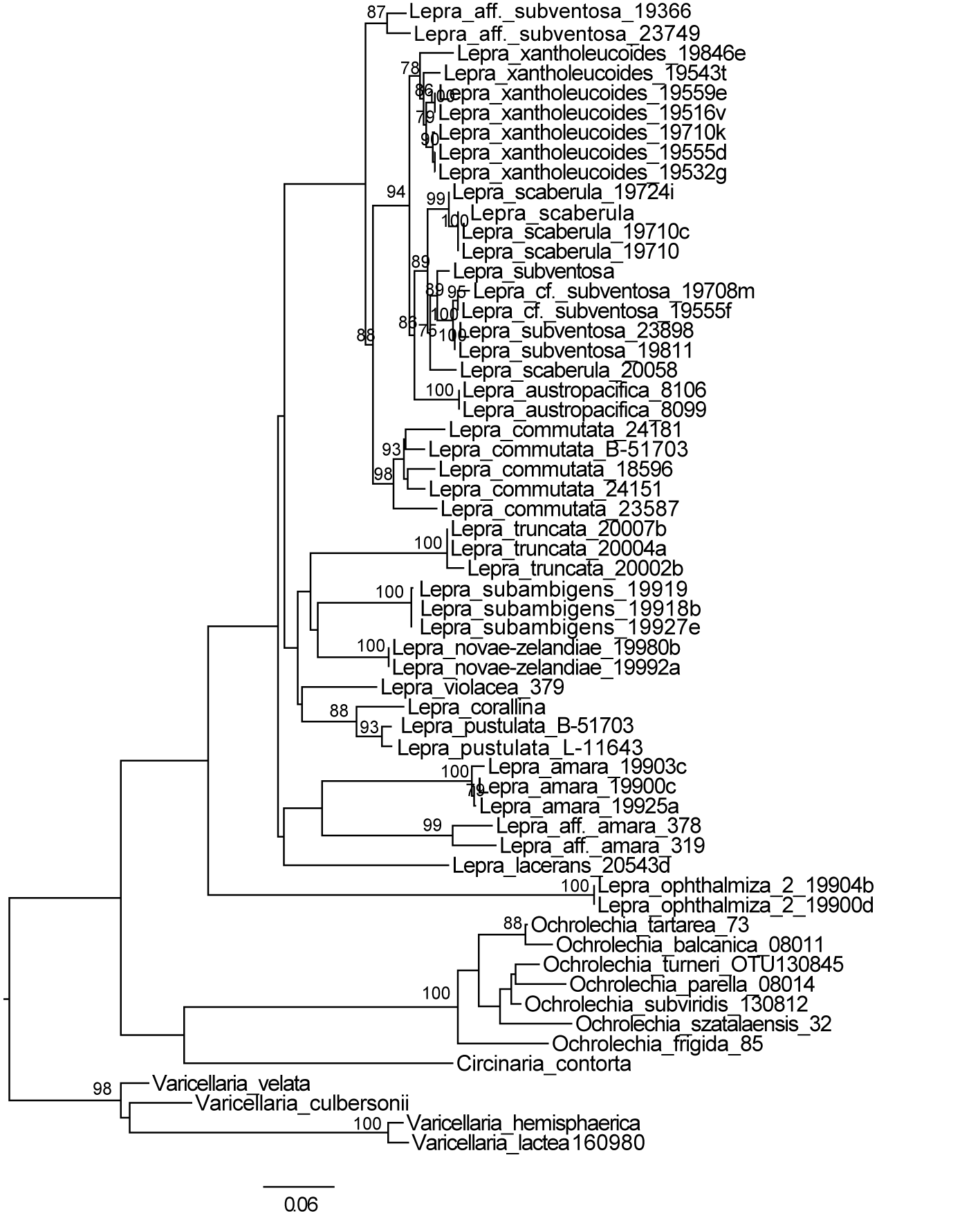

Supplement: S4 Fig — This is a RAxML tree based on MCM7 concatenated sequences. The numbers at each node represent bootstrap support value, and numbers lower than 50 not shown. Scale = 0.03 substitution per site. (TIF) [file pone.0180284.s004.tif]

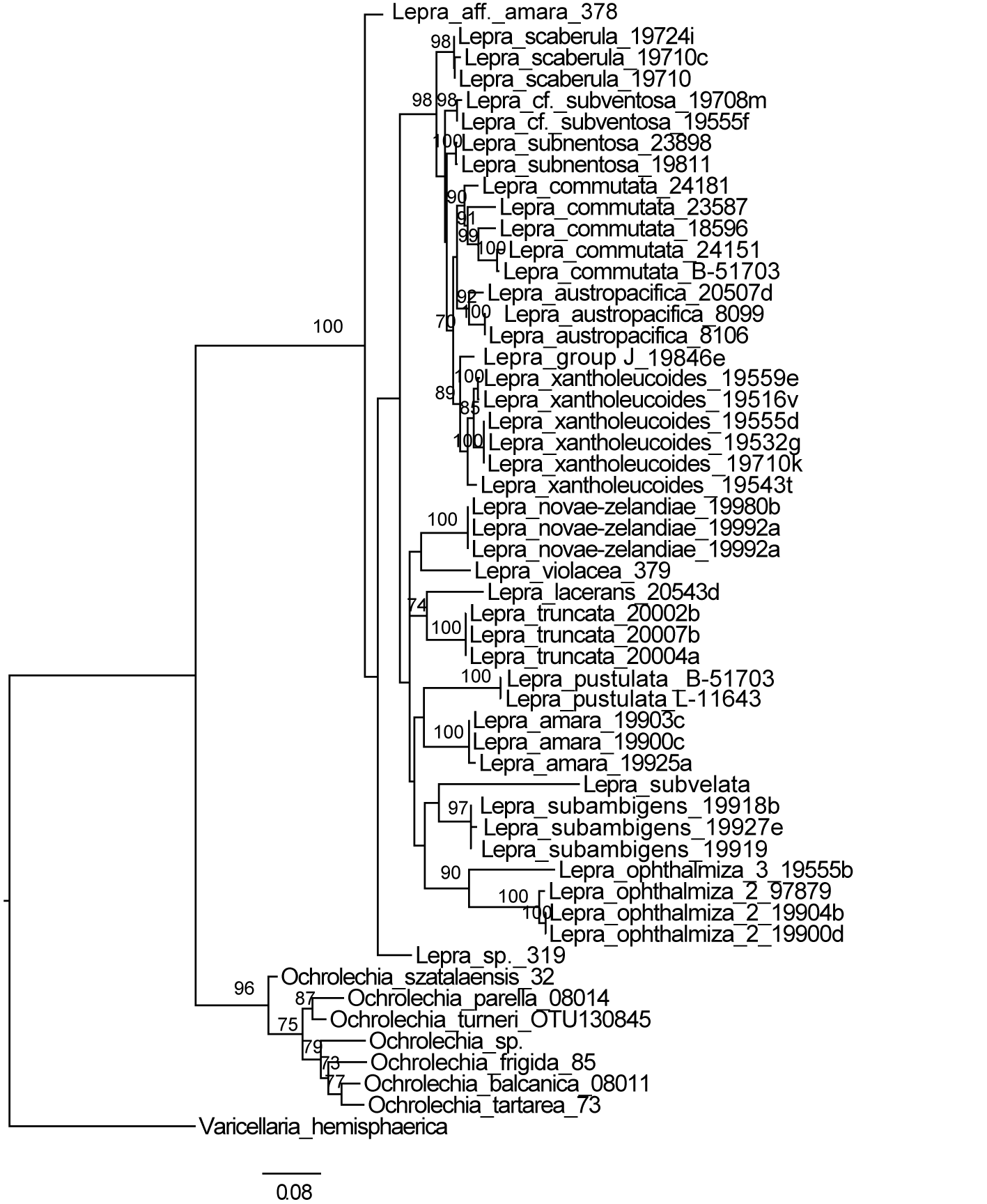

Supplement: S5 Fig — This is a RAxML tree based on EF1α sequences. The numbers at each node represent bootstrap support value, and numbers lower than 50 not shown. Scale = 0.03 substitution per site. (TIF) [file pone.0180284.s005.tif]

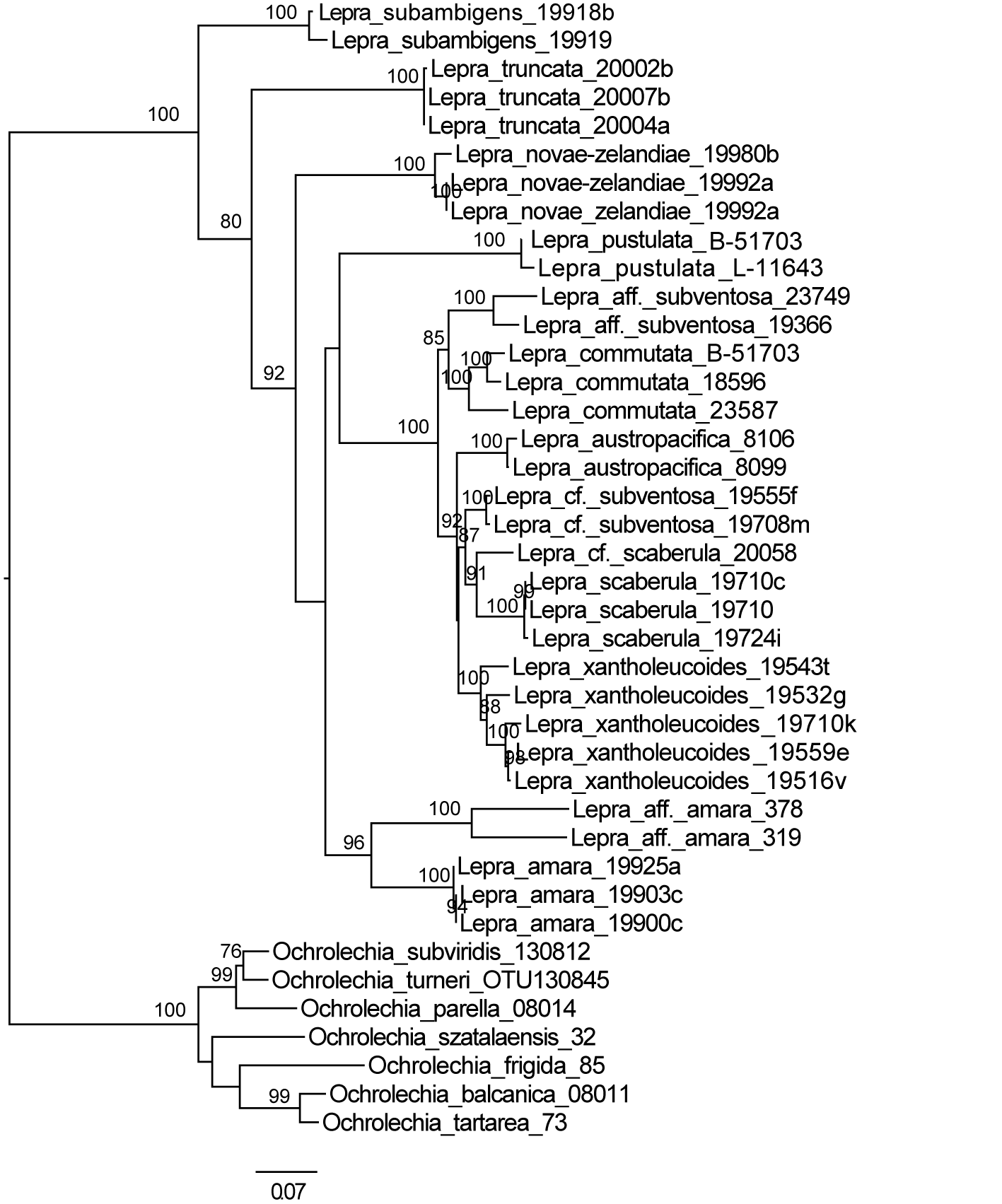

Supplement: S6 Fig — This is a RAxML tree based on TSR1sequences. The numbers at each node represent bootstrap support value, and numbers lower than 50 not shown. Scale = 0.03 substitution per site. (TIF) [file pone.0180284.s006.tif]
