# Supplementary material for: Circumscription of the genus Lepra, a recently resurrected genus to accommodate the “Variolaria”-group of Pertusaria sensu lato (Pertusariales, Ascomycota)
Source: PLoS One. 2017 Jul 11;12(7):e0180284. doi: 10.1371/journal.pone.0180284 (PMC5507398; doi:10.1371/journal.pone.0180284)
Supplement: S1 Table — (DOCX) [file pone.0180284.s007.docx]

**S1 Table. Specimens used for DNA extraction and samples from GenBank in this study**

| **Species** | **Synonym** | **Voucher/collection locality** | **LSU** | **MtSSU** | **RPB1** | **MCM7** | **EF1α** | **TSR1** |
| --- | --- | --- | --- | --- | --- | --- | --- | --- |
| *Circinaria contorta** |  |  | DQ986782 | DQ986876 | DQ986852 | - | - | - |
| *Circinaria contorta** |  |  | - | - | - | GU980989 | - | - |
| *Circinaria hispida** |  |  | DQ780305 | DQ780273 | - | - | - | - |
| *Circinaria hispida** |  |  | - | HM060722 | - | - | - | - |
| *Circinaria hispida** |  |  | - | - | DQ870933 | - | - | - |
| *Lepra albescens** | *Pertusaria albescens* |  | AF329176 | AF329175 | DQ870964 | - | - | - |
| *Lepra amara* | *Pertusaria amara* | Lumbsch 19903c (F), USA | MF109181 | MF109133 | MF189781 | MF189738 | MF279153 | MF189827 |
| *Lepra amara* | *Pertusaria amara* | Lumbsch 19925a (F), USA | - | MF109134 | MF189782 | MF189739 | MF189726 | MF189828 |
| *Lepra amara* | *Pertusaria amara* | Lumbsch 19900c (F), USA | MF109182 | MF109135 | MF189783 | MF189740 | MF279154 | MF189829 |
| *Lepra amara** | *Pertusaria amara* |  | AF274101 | - | - | - | - | - |
| *Lepra amara** | *Pertusaria amara* |  | JN941359 | - | JN992652 | - | - | - |
| *Lepra amara** | *Pertusaria amara* |  | - | - | KJ766829 | - | - | - |
| *Lepra amara** | *Pertusaria amara* |  | KJ766623 | - | - | - | - | - |
| *Lepra amara** | *Pertusaria amara* |  | - | AY300900 | - | - | - | - |
| *Lepra amara** | *Pertusaria amara* |  | - | - | DQ870965 | - | - | - |
| *Lepra amara** | *Pertusaria amara* |  | - | - | DQ973048 | - | - | - |
| *Lepra* aff. *amara* | *Pertusaria* aff. *amara* | Divakar 319 (MAF), Japan | MF109212 | MF109168 | MF189815 | MF189770 | MF279178 | MF189851 |
| *Lepra* aff. *amara* | *Pertusaria* aff. *amara* | Divakar 318 (MAF), Japan | MF109213 | MF109169 | - | - | - | - |
| *Lepra* aff. *amara* | *Pertusaria* aff. *amara* | Divakar 378 (MAF), Japan | MF109214 | MF109170 | MF189816 | MF189771 | MF279179 | MF189852 |
| *Lepra aspergilla** | *Pertusaria aspergilla* |  | - | AY567986 | - | - | - | - |
| *Lepra austropacifica* | *Pertusaria* aff. *scaberula* | Lumbsch 8106 (F), New Caledonia | MF109216 | MF109172 | MF189818 | MF189772 | MF189736 | MF189853 |
| *Lepra austropacifica* | *Pertusaria* aff. *scaberula* | Lumbsch 8099 (F), New Caledonia | MF109217 | MF109173 | MF189819 | MF189773 | MF189737 | MF189854 |
| *Lepra austropacifica* | *Pertusaria* aff. *scaberula* | Lumbsch 20507d (F), Fiji | MF109211 | MF109167 | MF189814 | - | MF279177 | - |
| *Lepra commutata* | *Pertusaria copiosa* | Lendemer B-51703 (NYBG), USA | MF109183 | MF109136 | - | MF189741 | MF279155 | MF189830 |
| *Lepra commutata* | *Pertusaria commutata* | Flakus 24151 (KRAM), Bolivia | MF109184 | MF109138 | MF189785 | MF189743 | MF279156 | - |
| *Lepra commutata* | *Pertusaria commutata* | Flakus 24181 (KRAM), Bolivia | MF109187 | MF109137 | MF189784 | MF189742 | MF189727 | - |
| *Lepra commutata* | *Pertusaria commutata* | Flakus 18596 (KRAM), Bolivia | MF109185 | MF109141 | MF189788 | MF189746 | MF279159 | MF189833 |
| *Lepra commutata* | *Pertusaria commutata* | Flakus 23587 (KRAM), Bolivia | MF109190 | MF109142 | MF189789 | MF189747 | - | MF189834 |
| *Lepra corallina** | *Pertusaria corallina* |  | AY300850 | AY300901 | - | - | - | - |
| *Lepra corallina** | *Pertusaria corallina* |  | - | DQ780286 | - | - | - | - |
| *Lepra corallina** | *Pertusaria corallina* |  | - | - | DQ870967 | GU980997 | - | - |
| *Lepra corallophora** | *Pertusaria corallophora* |  | DQ780315 | DQ780284 | - | - | - | - |
| *Lepra corallophora** | *Pertusaria corallophora* |  | DQ780316 | DQ780285 | DQ870969 | - | - | - |
| *Lepra dactylina** | *Pertusaria dactylina* |  | DQ782907 | - | DQ782828 | - | DQ782899 | - |
| *Lepra dactylina** | *Pertusaria dactylina* |  | - | DQ912307 | - | - | - | - |
| *Lepra dactylina** | *Pertusaria dactylina* |  | - | DQ972973 | - | - | - | - |
| *Lepra dactylina** | *Pertusaria dactylina* |  | - | AY567985 | - | - | - | - |
| *Lepra erythrella** | *Pertusaria erythrella* |  | AF274100 | AF431958 | - | - | - | - |
| *Lepra excludens** | *Pertusaria excludens* |  | DQ780318 | - | - | - | - | - |
| *Lepra excludens** | *Pertusaria excludens* |  | EF489944 | - | - | - | - | - |
| *Lepra excludens** | *Pertusaria excludens* |  | - | AY567987 | - | - | - | - |
| *Lepra* aff. *subventosa* | *Pertusaria* aff. *subventosa* | Flakus 19366 (PAN), Bolivia | MF109188 | MF109139 | MF189786 | MF189744 | - | MF189831 |
| *Lepra* aff. *subventosa* | *Pertusaria* aff. *subventosa* | Flakus 23749 (PAN), Bolivia | MF109189 | MF109140 | MF189787 | MF189745 | - | MF189832 |
| *Lepra lacerans* | *Pertusaria lacerans* | Lumbsch 20543d (F), Fiji | MF109205 | MF109158 | MF189805 | MF189763 | MF279171 | - |
| *Lepra lactescens** | *Pertusaria lactescens* |  | AY568004 | - | - | - | - | - |
| *Lepra mammosa** | *Pertusaria mammosa* |  | AY212831 | AY212854 | - | - | - | - |
| *Lepra mammosa** | *Pertusaria mammosa* |  | - | - | DQ870974 | - | - | - |
| *Lepra novae-zelandiae* | *Pertusaria novae-zelandiae* | Lumbsch 19992a (F), Australia | MF109206 | MF109159 | MF189806 | - | MF279172 | MF189846 |
| *Lepra novae-zelandiae* | *Pertusaria novae-zelandiae* | Lumbsch 19980b (F), Australia | MF109207 | MF109160 | MF189807 | MF189764 | MF279173 | MF189847 |
| *Lepra novae-zelandiae* | *Pertusaria novae-zelandiae* | Lumbsch 19992a (F), Australia | MF109208 | MF109161 | MF189808 | MF189765 | MF279174 | MF189848 |
| *Lepra ophthalmiza**1 | *Pertusaria ophthalmiza* |  | AY568006 | AY567993 | DQ870976 | - | - | - |
| *Lepra ophthalmiza*2 | *Pertusaria ophthalmiza* | Lumbsch 19555b (F), Kenya | MF109215 | MF109171 | MF189817 | - | MF279180 | - |
| *Lepra ophthalmiza*3 | *Pertusaria ophthalmiza* | Lumbsch 19900d (F), USA | MF109226 | MF109162 | MF189809 | MF189766 | MF279175 | - |
| *Lepra ophthalmiza*3 | *Pertusaria ophthalmiza* | Wetmore 97879 (UMN), USA | MF109227 | MF109163 | MF189810 | - | MF189733 | - |
| *Lepra ophthalmiza*3 | *Pertusaria ophthalmiza* | Lumbsch 19904b (F), USA | MF109225 | MF109164 | MF189811 | MF189767 | MF189734 | - |
| *Lepra panyrga** | *Pertusaria panyrga* |  | DQ780327 | AY567994 | DQ870977 | - | - | - |
| *Lepra pustulata* | *Variolaria pustulata* | Lendemer L-11643 (NYBG), USA | MF109209 | MF109165 | MF189812 | MF189768 | MF279176 | MF189849 |
| *Lepra pustulata* | *Variolaria pustulata* | Lendemer B-51703 (NYBG), USA | MF109210 | MF109166 | MF189813 | MF189769 | MF189735 | MF189850 |
| *Lepra scaberula* | *Pertusaria scaberula* | Lumbsch 19710 (F), India | MF109191 | MF109143 | MF189790 | MF189748 | MF279160 | MF189835 |
| *Lepra scaberula* | *Pertusaria scaberula* | Flakus 20058 (KRAM), Bolivia | MF109192 | MF109144 | MF189791 | MF189749 | - | MF189836 |
| *Lepra scaberula* | *Pertusaria scaberula* | Lumbsch 19710c (F), India | MF109193 | MF109145 | MF189792 | MF189750 | MF279161 | MF189837 |
| *Lepra scaberula* | *Pertusaria scaberula* | Lumbsch 19724i (F), India | MF109194 | MF109146 | MF189793 | MF189751 | MF189728 | MF189838 |
| *Lepra scaberula** | *Pertusaria scaberula* |  | AF274099 | AF431959 | - | - | - | - |
| *Lepra scaberula** | *Pertusaria scaberula* |  | - | - | DQ870980 | - | - | - |
| *Lepra scaberula** | *Pertusaria scaberula* |  | - | - | - | GU981003 | - | - |
| *Lepra subambigens* | *Pertusaria subambigens* | Lumbsch 19927e (F), USA | MF109218 | MF109174 | MF189820 | MF189774 | MF279181 | - |
| *Lepra subambigens* | *Pertusaria subambigens* | Lumbsch 19918b (F), USA | MF109219 | MF109175 | MF189821 | MF189775 | MF279182 | MF189855 |
| *Lepra subambigens* | *Pertusaria subambigens* | Lumbsch 19919 (F), USA | MF109220 | MF109176 | MF189822 | MF189776 | MF279183 | MF189856 |
| *Lepra subventosa* | *Pertusaria subventosa* | Flakus 23898 (KRAM), Bolivia | MF109195 | MF109147 | MF189794 | MF189752 | MF279162 | - |
| *Lepra subventosa* | *Pertusaria subventosa* | Flakus 19811 (KRAM), Bolivia | MF109196 | MF109148 | MF189795 | MF189753 | MF279163 | - |
| *Lepra subventosa** | *Pertusaria subventosa* |  | AY300854 | AY300905 | - | - | - | - |
| *Lepra subventosa** | *Pertusaria subventosa* |  | - | DQ780302 | - | - | - | - |
| *Lepra subventosa** | *Pertusaria subventosa* |  | - | - | DQ870981 | - | - | - |
| *Lepra subventosa** | *Pertusaria subventosa* |  | - | - | - | GU981004 | - | - |
| *Lepra* cf. *subventosa* | *Pertusaria* cf. *subventosa* | Lumbsch 19708m (F), India | MF109197 | MF109149 | MF189796 | MF189754 | MF279164 | MF189839 |
| *Lepra* cf. *subventosa* | *Pertusaria* cf. *subventosa* | Lumbsch 19555f (F), Kenya | MF109198 | MF109150 | MF189797 | MF189755 | MF279165 | MF189840 |
| *Lepra truncata* | *Pertusaria truncata* | Lumbsch 20004a (F), Australia | MF109221 | MF109177 | MF189823 | MF189777 | MF279184 | MF189857 |
| *Lepra truncata* | *Pertusaria truncata* | Lumbsch 20007b (F), Australia | MF109222 | MF109178 | MF189824 | MF189778 | MF279185 | MF189858 |
| *Lepra truncata* | *Pertusaria truncata* | Lumbsch 20002b (F), Australia | MF109223 | MF109179 | MF189825 | MF189779 | MF279186 | MF189859 |
| *Lepra violacea* | *Pertusaria violacea* | Divakar 379 (MAF), Japan | MF109224 | MF109180 | MF189826 | MF189780 | MF279187 | - |
| *Lepra xantholeucoides* | *Pertusaria kinigiensis* | Lumbsch 19543t (F), Kenya | MF109199 | MF109151 | MF189798 | MF189756 | MF279166 | MF189841 |
| *Lepra xantholeucoides* | *Pertusaria kinigiensis* | Lumbsch 19516v (F), Kenya | MF109200 | MF109152 | MF189799 | MF189757 | MF189729 | MF189842 |
| *Lepra xantholeucoides* | *Pertusaria kinigiensis* | Lumbsch 19532g (F), Kenya | MF109186 | MF109153 | MF189800 | MF189758 | MF189730 | MF189843 |
| *Lepra xantholeucoides* | *Pertusaria kinigiensis* | Lumbsch 19555d (F), Kenya | MF109201 | MF109154 | MF189801 | MF189759 | MF279167 | - |
| *Lepra xantholeucoides* | *Pertusaria kinigiensis* | Lumbsch 19559e (F), Kenya | MF109202 | MF109155 | MF189802 | MF189760 | MF189731 | MF189844 |
| *Lepra xantholeucoides* | *Pertusaria xantholeucoides* | Lumbsch 19846e (F), Fiji | MF109203 | MF109156 | MF189803 | MF189761 | MF279170 | - |
| *Lepra xantholeucoides* | *Pertusaria xantholeucoides* | Lumbsch 19710k (F), India | MF109204 | MF109157 | MF189804 | MF189762 | MF189732 | MF189845 |
| *Lobothallia radiosa** |  |  | KJ766596 | KJ766430 | KJ766870 | - | - | - |
| *Lobothallia radiosa** |  |  | DQ780306 | - | - | - | - | - |
| *Pertusaria californica** |  |  | - | - | GU981010 | - | - | - |
| *Pertusaria carneopallida** |  |  | GU980987 | - | GU981011 | - | - | - |
| *Pertusaria* cf. *quartans** |  |  | DQ780333 | DQ780298 | - | - | - | - |
| *Pertusaria cineraria** |  |  | - | AY567983 | - | - | - | - |
| *Pertusaria coccodes** |  |  | AF279295 | - | - | - | - | - |
| *Pertusaria coccodes** |  |  | - | AY567984 | - | - | - | - |
| *Pertusaria coronata** |  |  | DQ780314 | - | - | - | - | - |
| *Pertusaria coronata** |  |  | AY300851 | AY300902 | - | - | - | - |
| *Pertusaria dehiscens** |  |  | DQ780317 | - | - | - | - | - |
| *Pertusaria flavicans** |  |  | DQ780320 | DQ780287 | - | - | - | - |
| *Pertusaria flavicunda** |  |  | AF279299 | AF381562 | - | - | - | - |
| *Pertusaria flavida** |  |  | AY568003 | - | - | - | - | - |
| *Pertusaria flavida** |  |  | DQ780319 | - | - | - | - | - |
| *Pertusaria graphica** |  |  | DQ780323 | DQ780290 | - | - | - | - |
| *Pertusaria hermaka** |  |  | DQ780334 | DQ780299 | JX101872 | - | - | -- |
| *Pertusaria hermaka** |  |  | - | - | KC222186 | - | - | - |
| *Pertusaria hymenea** |  |  | - | AY567988 | - | - | - | - |
| *Pertusaria kalelae** |  |  | AF279298 | - | - | - | - | - |
| *Pertusaria kalelae** |  |  | - | AY567989 | - | - | - | - |
| *Pertusaria lactescens** |  |  | - | AY567990 | - | - | - | - |
| *Pertusaria laeviganda** |  |  | DQ780324 | DQ780291 | - | - | - | - |
| *Pertusaria lecanina** |  |  | AF279296 | - | - | - | - | - |
| *Pertusaria lecanina** |  |  | - | AY567991 | - | - | - | - |
| *Pertusaria leioplaca** |  |  | AY300852 | AY300903 | - | - | - | - |
| *Pertusaria mesotropa** |  |  | DQ780325 | DQ780292 | - | - | - | - |
| *Pertusaria oculata** |  |  | AY568005 | AY567992 | - | - | - | - |
| *Pertusaria paramerae** |  |  | DQ780326 | DQ780293 | - | - | - | - |
| *Pertusaria paramerae** |  |  | - | HM176597 | - | - | - | - |
| *Pertusaria paramerae** |  |  | - | GU980980 | GU981012 | - | - | - |
| *Pertusaria pertracta** |  |  | DQ780321 | DQ780288 | - | - | - | - |
| *Pertusaria pertracta** |  |  | DQ780322 | DQ780289 | - | - | - | - |
| *Pertusaria pertusa** |  |  | JN941360 | - | JN992653 | - | - | - |
| *Pertusaria pertusa** |  |  | - | - | KJ766877 | - | - | - |
| *Pertusaria pertusa** |  |  | KP794960 | KR017385 | - | - | - | - |
| *Pertusaria pertusa** |  |  | AF279300 | AF381565 | - | - | - | - |
| *Pertusaria plittiana** |  |  | DQ780328 | DQ780294 | - | - | - | - |
| *Pertusaria pupillaris** |  |  | AY568007 | AY567995 | - | - | - | - |
| *Pertusaria pustulata** |  |  | DQ780329 | DQ780295 | - | - | - | - |
| *Pertusaria pustulata** |  |  | DQ780330 | - | - | - | - | - |
| *Pertusaria pustulata** |  |  | DQ780332 | DQ780297 | - | - | - | - |
| *Pertusaria pustulata** |  |  | DQ780331 | - | GU981013 | - | - | - |
| *Pertusaria rhodiensis** |  |  | AY568008 | AY567996 | - | - | - | - |
| *Pertusaria rupicola* var. *coralloidea** |  |  | AY300853 | AY300904 | - | - | - | - |
| *Pertusaria signyae** |  |  | - | AY567997 | - | - | - | - |
| *Pertusaria subobductans** |  |  | DQ780336 | DQ780301 | - | - | - | - |
| *Pertusaria subobductans** |  |  | - | DQ780300 | - | - | - | - |
| *Pertusaria subvelata** |  |  | KR017227 | KR017389 | - | - | KR017581 | - |
| *Pertusaria subverrucosa** |  |  | DQ780335 | FJ941884 | FJ941904 | - | - | - |
| *Pertusaria tejocotensis** |  |  | AF279301 | AF381566 | - | - | - | - |
| *Pertusaria variolosa** |  |  | DQ780338 | - | - | - | - | - |
| *Pertusaria variolosa** |  |  | DQ780339 | - | - | - | - | - |
| *Pertusaria variolosa** |  |  | DQ780337 | - | - | - | - | - |
| *Pertusaria werneriana** |  |  | AY300856 | AY300907 | - | - | - | - |
| *Pertusaria xanthoplaca** |  |  | DQ780340 | - | - | - | - | - |
| *Varicellaria culbersonii** |  |  | JX101871 | JX101873 | JX101875 | JX101874 | - | - |
| *Varicellaria hemisphaerica** |  |  | AF381556 | AF381563 | - | GU980998 | DQ902343 | - |
| *Varicellaria hemisphaerica** |  |  | - | DQ973000 | DQ902341 | - |  | - |
| *Varicellaria lactea** |  |  | AF381557 | AF381564 | DQ870971 | GU981000 | - | - |
| *Varicellaria rhodocarpa** |  |  | AF381559 | AF381569 | - | - | - | - |
| *Varicellaria velata** |  |  | AY300855 | AY300906 | DQ870982 | - | - | - |
| *Varicellaria velata** |  |  | - | GU980981 | - | GU981005 | - | - |

Notes: The sequences of species marked by * were downloaded from GenBank; missing sequences were indicated by dashes.
